# Supplementary material for: Comparative genomics provides new insights into the diversity, physiology, and sexuality of the only industrially exploited tremellomycete: Phaffia rhodozyma
Source: BMC Genomics. 2016 Nov 9;17:901. doi: 10.1186/s12864-016-3244-7 (PMC5103461; doi:10.1186/s12864-016-3244-7)
Supplement: Additional file 6: — List of orphan genes with links to PFAM (related to Additional file 1: Table S1). (ZIP 1428 kb) [file 12864_2016_3244_MOESM6_ESM.zip › PFAM_UNIQUE/G02844_P.html]

#                                                                            --- full sequence --- -------------- this domain -------------   hmm coord   ali coord   env coord
  
# target name        accession   tlen query name           accession   qlen   E-value  score  bias   #  of  c-Evalue  i-Evalue  score  bias  from    to  from    to  from    to  acc description of target
  
#------------------- ---------- ----- -------------------- ---------- ----- --------- ------ ----- --- --- --------- --------- ------ ----- ----- ----- ----- ----- ----- ----- ---- ---------------------
  
G02844\_P             -            588 LRR\_4                PF12799.2     44    0.0015   16.8   6.4   1   4    0.0021      0.51    8.7   0.1     1    30   170   198   157   200 0.85 - Leucine Rich repeats (2 copies)  
G02844\_P             -            588 LRR\_4                PF12799.2     44    0.0015   16.8   6.4   2   4      0.42        99    1.4   0.0    18    35   324   341   316   343 0.77 - Leucine Rich repeats (2 copies)  
G02844\_P             -            588 LRR\_4                PF12799.2     44    0.0015   16.8   6.4   3   4      0.18        43    2.6   0.1    22    31   354   363   350   364 0.88 - Leucine Rich repeats (2 copies)  
G02844\_P             -            588 LRR\_4                PF12799.2     44    0.0015   16.8   6.4   4   4      0.11        26    3.3   0.1    18    32   461   475   454   476 0.83 - Leucine Rich repeats (2 copies)  
G02844\_P             -            588 LRR\_8                PF13855.1     61      0.08   11.4   8.3   1   4     0.024       6.4    5.3   0.1    19    52   164   198   156   199 0.72 - Leucine rich repeat  
G02844\_P             -            588 LRR\_8                PF13855.1     61      0.08   11.4   8.3   2   4     0.028       7.7    5.0   0.1    16    58   296   339   295   341 0.84 - Leucine rich repeat  
G02844\_P             -            588 LRR\_8                PF13855.1     61      0.08   11.4   8.3   3   4       1.2   3.2e+02   -0.1   0.1    23    32   354   363   351   364 0.77 - Leucine rich repeat  
G02844\_P             -            588 LRR\_8                PF13855.1     61      0.08   11.4   8.3   4   4      0.26        70    2.0   0.2    45    57   463   475   455   476 0.85 - Leucine rich repeat
